# Psychological side effects of antipsychotic medication after remission from first-episode psychosis: a HAMLETT ecological momentary assessment study

**DOI:** 10.1017/S003329172510216X

**Published:** 2025-10-29

**Authors:** Matej Djordjevic, Shiral S. Gangadin, Lieuwe de Haan, Hannah E. Jongsma, Priscilla P. Oomen, Claudia J. P. Simons, Machteld Marcelis, Mark Nijland, Marieke J. H. Begemann, Iris E. C. Sommer, Martijn J. Kikkert, Wim Veling

**Affiliations:** 1 University of Groningen, University Medical Center Groningen, University Center for Psychiatry, Rob Giel Research Center, Groningen, The Netherlands; 2 Center for Clinical Neuroscience and Cognition, University of Groningen, University Medical Center Groningen, Groningen, The Netherlands; 3 Department of Early Psychosis, Amsterdam University Medical Center, Amsterdam, The Netherlands; 4 Center for Transcultural Psychiatry Veldzicht, Balkbrug, The Netherlands; 5 Behavioural Science Institute, Radboud University, Nijmegen, The Netherlands; 6 Department of Psychiatry and Neuropsychology, Mental Health and Neuroscience Research Institute, Maastricht University, Maastricht, The Netherlands; 7 GGzE, Institute for Mental Health Care Eindhoven, Eindhoven, The Netherlands; 8 Department of Research, Arkin Mental Health Care, Amsterdam, The Netherlands

**Keywords:** first-episode psychosis, antipsychotic medication, D_2_ affinity, side effects, ecological momentary assessment, experience sampling methodology, schizophrenia spectrum disorder, early psychosis, psychopharm acology

## Abstract

**Background:**

Evidence on psychological side effects (PSEs) of antipsychotic medication after remission from first-episode psychosis (FEP), and their momentary impact on daily life, is limited. This study examined how Dopamine-2 (D_2_) affinity and antipsychotic dosage relate to momentary PSEs.

**Methods:**

This ecological momentary assessment (EMA) study included baseline data from 56 participants in the ongoing Handling Antipsychotic Medication: Long-term Evaluation of Targeted Treatment (HAMLETT) trial. Momentary mental states indicative of reduced affect intensity, stability, and variability, as well as avolition and mental fatigue, were assessed 10×/day for eight days (N = 3,005 data points). Since these PSEs may result from D_2_-receptor actions, antipsychotics were classified by receptor affinity and mechanism of action. Multilevel mixed-effects regression models examined serial cross-sectional associations between D_2_ affinity or dosage and concurrent PSEs, both overall and separately for mornings, daytimes, and evenings.

**Results:**

Higher antipsychotic dosages were associated with reduced affect variability (Beta [B] = −1.40 [95% confidence interval [CI]: −2.52; −0.29]) and decreased positive affect stability (B = 0.23 [95% CI: 0.04; 0.42]) and intensity (B = −1.11 [95% CI: −1.97; −0.24]). The latter was also associated with the use of high-affinity D_2_ antagonists versus partial D_2_ agonists (B = 12.98 [95% CI: 2.43; 23.53]) and versus low-affinity D_2_ antagonists (B = 10.04 [95% CI: 0.59; 19.49]). Other PSEs were not associated with D_2_ affinity/dosage. Results were relatively consistent across daytimes.

**Conclusions:**

Higher antipsychotic dosage and high-affinity D_2_ antagonists were associated with decreased positive affect after remission from FEP, which may partly drive the frequently reported blunting of emotional experience.

## Background

Pharmaceutical agents acting primarily on Dopamine-2 (D_2_) receptors (‘antipsychotics’) are a cornerstone of the current medical treatment for psychotic disorders. However, their side effects often pose a significant burden on patients (Bjornestad et al., 2020; Read & Sacia, 2020; Thompson et al., 2020). Ecologically valid insights into different side effects across various social contexts are important for well-informed decision-making between clinicians and patients. However, the currently available evidence from side effects as they are experienced in daily life is scarce (Bos, Schoevers, & aan het Rot, 2015).

There is robust evidence (Ceraso et al., 2020; Haddad, Das, Keyhani, & Chaudhry, 2012; Zhang et al., 2013) showing that antipsychotic medication is effective for managing acute symptoms of psychosis. Most patients state that pharmaceutical intervention during the acute phase improved their recovery process (Bjornestad et al., 2020; Thompson et al., 2020). However, once acute psychotic symptoms abate, side effects of antipsychotic medication can become more cumbersome (Bjornestad et al., 2020; Read & Sacia, 2020; Thompson et al., 2020) and fuel the frequently expressed wish (Kahn et al., 2008) to discontinue or taper the medication earlier than currently recommended in various national guidelines (NICE, 2014; Orygen, 2010; Veling et al., 2017). Qualitative research shows that, in addition to somatic side effects including extrapyramidal motor symptoms and metabolic disturbances, patients taking antipsychotic medication can experience psychological side effects (PSEs) such as blunted emotional experience, reduced motivation, difficulty concentrating, or tiredness (i.e. mental fatigue) (Bjornestad et al., 2020; de Haan et al., 2000, 2003; Read & Sacia, 2020; Thompson et al., 2020; Wolters, Knegtering, Wiersma, & Van Den Bosch, 2003). These PSEs have previously been labeled as ‘secondary negative symptoms’ (Kaar, Natesan, McCutcheon, & Howes, 2020; Kirschner, Aleman, & Kaiser, 2017; Mas et al., 2013), since they resemble the ‘primary negative symptoms’ inherent to schizophrenia spectrum disorders (Correll & Schooler, 2020). They may generally be explained by suppression of dopamine activity or simultaneous action on other neurotransmitter systems (Kaar et al., 2020; Kirschner et al., 2017; Sabe, Zhao, Crippa, & Kaiser, 2021). Whereas PSEs appear to be common (e.g. nonprimary negative symptoms were present in 45% of 1,452 people with psychotic disorders (Bobes et al., 2010)), they remain largely underrepresented in the current literature on side effects of antipsychotic medication (Longden & Read, 2016) and have rarely been investigated in the flow of daily life (Bos et al., 2015). The resulting lack of ecologically valid insights into people’s emotional experiences under antipsychotic treatment impedes the understanding of the full side effect breadth, while this is crucial for providing optimal clinical advice.

Ecological momentary assessment (EMA) (or the experience sampling method [ESM]) is a method to study daily life emotions, experiences, and social context (Myin-Germeys et al., 2009). Compared with retrospective clinical questionnaires, EMA allows for in-the-moment yet dynamic investigations (Granholm et al., 2019; Mote & Fulford, 2020; Wright et al., 2021), while mitigating the risk of peak-end and recall bias (Horwitz, Zhao, & Sen, 2023; Urban, Charles, Levine, & Almeida, 2018). The real-time assessments of momentary mental states such as positive and negative affect (PA/NA) are well suited to capture experiences that may be indicative of the PSEs described above (Bjornestad et al., 2020; Read & Sacia, 2020; Thompson et al., 2020; Wolters et al., 2003), for example, altered PA/NA intensity and variability as indicators of blunted emotional experience. They also allow for distinct investigations at various times of the day, which may have relevant implications regarding the potential impact PSEs might have on people’s daily life. For example, mental fatigue could be especially burdensome in the morning when people need to get ready for daytime activities (including work, college, appointments, or other obligations), while blunted emotional experience could interfere with activities that involve social interactions. To the best of our knowledge, so far only a few studies have employed EMA to examine associations between the use of antipsychotic medication and momentary affect (Lataster et al., 2011a, 2011b; Oomen, Simons, Broekmans-Madikrama, & Marcelis, 2024). One study suggests that increased D_2_ receptor occupancy in people with psychotic disorders taking high-affinity D_2_ antagonists is associated with decreased PA and increased NA (Lataster et al., 2011b). Another study found that switching to aripiprazole was associated with decreases in both PA and NA in six patients with schizophrenia (Lataster et al., 2011a). Insights into other PSEs are lacking. Moreover, those findings require replication and might be different for people who are in recent clinical remission from first-episode psychosis (FEP).

Our study aimed to analyze EMA data of people in clinical remission from FEP in order to investigate how D_2_ affinity and dosage of antipsychotic medication are associated with momentary PSEs throughout the day, that is, overall as well as separately in the beginning, during and at the end of the day.

Based on previous studies (de Beer et al., 2024; Lataster et al., 2011a, 2011b; Veerman, Schulte, & de Haan, 2017), we hypothesized that higher D_2_ affinity and higher dosage of antipsychotics are associated with increased levels of momentary blunted emotional experience, avolition, and mental fatigue, and that effect sizes are larger in the beginning of the day.

## Methods

### Study population

Data for the current study were collected between September 2017 and May 2023 through baseline assessments of the ongoing Handling Antipsychotic Medication: Long-term Evaluation of Targeted Treatment (HAMLETT) study (Begemann et al., 2020). HAMLETT is a multicenter, pragmatic, randomized controlled trial in the Netherlands evaluating the effect of early (dis-)continuation of antipsychotic medication on various outcomes in people who are in remission from FEP. Participants are between 16 and 60 years of age, in clinical remission (Andreasen et al., 2005) from FEP for 3–6 months, have sufficient command of the Dutch language, and have provided written informed consent. Exclusion criteria include dangerous or harmful behavior and coercive treatment with antipsychotic medication during FEP. Further details can be found in the published study protocol (Begemann et al., 2020).

At baseline, participants were invited to take part in the EMA add-on study until a pre-determined sample size was reached. Note that there was a temporary recruitment-pause due to COVID-19 and that not every participant has been invited to take part in the EMA add-on study. All study procedures were in line with local and international ethical standards, including the Declaration of Helsinki. Ethical approval was issued by the Medical Ethical Committee of the University Medical Center Groningen in the Netherlands (HAMLETT protocol number: NL62202.042.17, trial registration EudraCT number: 2017-002406-12).

### Measurements

Baseline assessments took place during a study visit in one of the three study centers, a home visit, or a study visit in one of the recruiting mental health care institutions, and were performed by trained researchers. The EMA included repeated self-report data collection over eight consecutive days.

Sociodemographic, premorbid, and general clinical information was collected through the Comprehensive Assessment of Symptoms and History (CASH) (Andreasen, Flaum, & Arndt, 1992), a semistructured clinical interview developed for schizophrenia spectrum disorders. The CASH includes information on substance use and the severity of symptoms during FEP based on five 6-point Likert items assessing the global severity of hallucinations, delusions, bizarre behavior, apathy, and anhedonia. Baseline clinical symptom severity was assessed with the Positive and Negative Syndrome Scale (PANSS) (Kay, Fiszbein, & Opler, 1987). Subjective well-being in the past week was assessed using the Subjective Well-being under Neuroleptics Scale (Naber, 1995), a 20-item self-report questionnaire with higher scores indicating better well-being.

Data on the type and dosage of antipsychotic medication were retrieved from the Dutch Foundation for Pharmaceutical Statistics *Stichting Farmaceutische Kengetallen (SFK)*. The SFK collects prescription data from >98% of community pharmacies in the Netherlands. In case SFK-data were unavailable, medication information was reported by the participant or their treating psychiatrist. We grouped the medications based on D_2_ receptor affinity (*K_i_* cut-off value = 20.0; Kaar et al., 2020) and mechanism of action (D_2_ antagonism vs. partial agonism) into the following categories:

1. Low-affinity antagonists: olanzapine and quetiapine
2. High-affinity antagonists: haloperidol, risperidone, and amisulpride
3. Partial agonists: aripiprazole and brexpiprazole

Dosages were converted into olanzapine equivalents according to the 95% effective dose method (Leucht et al., 2020).

#### Momentary psychological side effects of antipsychotic medication

EMA took place up to ten times per day for eight consecutive days. The questionnaire consisted of 37 items and should be completed within a few minutes. Prompts were sent within time blocks of 1:20 h, with time intervals of at least 30 minutes. The software environment was provided by RoQua (https://www.RoQua.nl). Participants were briefed about the EMA procedure by trained researchers and contacted to provide further instructions when they did not complete at least one questionnaire within the first two days. Responses were rated on a Visual Analogue Scale (VAS) of 1–100. In line with the current literature suggesting a response rate cut-off of one-third to ensure adequate validity of EMA (Delespaul, 1995), we excluded subjects with <27 completed questionnaires from the analyses. Questionnaires were also excluded when subjects took >20 minutes for completion.

EMA items (Table 1) were selected to assess momentary mental states indicative of PSEs. Lack of initiating social contact or failing to complete daily life activities were selected as proxies for *avolition* (Oorschot, Kwapil, Delespaul, & Myin-Germeys, 2009), while difficulty concentrating or tiredness (Wolters et al., 2003) were selected as proxies for *mental fatigue.* Similarly, established EMA items assessing PA/NA (Bell et al., 2023; Myin-Germeys & van Os, 2007; Reininghaus et al., 2016) intensity, stability, and variability were used to measure *blunted emotional experience*, in line with previous work (Bos, de Jonge, & Cox, 2019; Hermans et al., 2021; Lataster et al., 2011a, 2011b). PSEs were investigated overall as well as separately for different times of the day, that is, beginning of the day (first three prompts per day), daytime (prompts 4–7) and end of the day (last three prompts per day). Cronbach’s alpha and McDonald’s omega coefficients were calculated to assess the internal reliability of the item groupings, when appropriate (Table 1).

**Table 1.** EMA indicators of psychological side effects

Psychological side effects of antipsychotic medication	
Side effect	EMA indicators
Blunted emotional experience	*Intensity of positive affect (PA) and negative affect (NA)* PA entailed the following items: ‘I feel cheerful’; ‘I feel enthusiastic’; ‘I feel satisfied’ (VAS 1–100; α = 0.9; ω = 0.9) NA entailed the following items: ‘I feel irritated’; ‘I feel sad’; ‘I feel anxious’; ‘I feel restless’; ‘I feel tense’. (VAS 1–100; α = 0.9; ω = 0.9)
	*Stability of PA and NA* This entailed the magnitude of the difference between prompt-level intensity of PA (or NA) and within-person mean intensity of PA (or NA).
	*Variability of PA and NA* This entailed the magnitude of the difference between prompt-level intensity of PA and concurrent NA.
Avolition^a^	‘Since the last measurement, I took initiative to make social contact, for example, by starting a chat’. (VAS 1–100)
	‘Since the last measurement, I have done what I needed or wanted to do’. (VAS 1–100)
Mental fatigue	Sum score of the items ‘I feel tired’. (reversed) and ‘I can concentrate well’. (VAS 1–100; α = 0.7)

*Note*: Higher scores indicate increased affect intensity and variability and more initiative of social contact and completion of daily life activities but less affect stability and mental fatigue. α, Cronbach’s alpha coefficient; ω, McDonald’s omega coefficient.

a The first prompt of the day was excluded from analyses.

### Statistical analyses

First, we employed descriptive analyses to characterize the study sample and the distribution of PSE scores. This included the calculation of frequencies and percentages for categorical variables and means with standard deviations or medians with interquartile ranges for continuous variables. We also ran missing value analyses.

#### Associations between antipsychotic medication and momentary psychological side effects

We performed stepwise multilevel mixed effects linear regression analyses (maximum likelihood estimation [MLE]) to investigate serial cross-sectional associations between the D_2_ affinity and dosage of antipsychotic medication and momentary indicators of PSEs throughout the day. In a first step, we ran models including D_2_ affinity and dosage as the independent variables and PSEs as the dependent variable. When at least one of the predictors was statistically significant (at α = 0.05) in a given model, we added an interaction term between D_2_ affinity and dosage. When neither of the two predictors was statistically significant, we additionally ran two separate models including only D_2_ affinity or dosage, respectively, as the single independent variable. All models were adjusted for fixed effects of age, sex, tobacco, and cannabis use in the past month and symptom severity during FEP (CASH). Tobacco and cannabis use in the past month as well as symptom severity during FEP were included as potential confounders because they may be associated with PSEs of antipsychotic medication (Kumari & Postma, 2005; Rashmi et al., 2016). Intraclass correlation coefficients (ICCs) were calculated to assess the degree of interindividual variation in the associations. Analyses were conducted overall as well as separately for different times of the day, that is, in the beginning, during, and at the end of the day. For all analyses, we used Stata Statistical Software: Release 18 (StataCorp., 2021).

## Results

In total, 85 out of 453 HAMLETT participants took part in the EMA add-on study. At baseline, 56 (66%) of those participants completed >26 EMA questionnaires and were currently taking antipsychotic medication, yielding a total of 3,005 questionnaires for our analyses. Thirteen participants (23%) were using psychotropic agents next to the antipsychotic, that is, benzodiazepines, anticholinergics, lithium, stimulants, or an SSRI/SNRI. The distribution of antipsychotic prescriptions was relatively equally spread (Table 2) and SFK data were available for 43 participants (77%). The average symptom severity (PANSS) at baseline reflects the HAMLETT inclusion criteria (i.e. being in clinical remission) as scores were generally low, especially in the positive subdomain (Mean [M] = 8.7, standard deviation [SD] = 2.5, Table 2). The global symptom severity during the FEP as, retrospectively, assessed with the CASH was also relatively low (M = 11.8, SD = 4.5, Table 2). Thirteen participants (23%) had at least one non-psychotic comorbid psychiatric diagnosis. The average EMA response rate was similar throughout the day and lower at night (66.2% between 6 and 13 h, 69.8% between 13 and 18 h, 66.8% between 18 and 24 h, 41.0% between 0 and 6 h), the average completion time per questionnaire was 2:32 min. Missing value analyses showed that there were no variables with >5% incomplete data (Table 3).

**Table 2.** Sociodemographic and clinical sample characteristics (*N* = 56)

Characteristic	
**Age**, mean (standard deviation), M (SD)	28.4 (9.7)
**Sex**, frequency (percentage), *N* (%)	
Female	21 (37.5)
Male	35 (62.5)
**Substance use in the past month**, *N* (%)	
Tobacco	19 (33.9)
Cannabis^a^	8 (14.8)
**Symptom severity during the FEP (CASH)**, M (SD)	11.8 (4.5)
**Symptom severity at study baseline (PANSS)**, M (SD)	
Total	42.2 (9.6)
Positive symptom domain	8.7 (2.5)
Negative symptom domain	11.6 (4.0)
General symptom domain	21.9 (5.0)
**Subjective well-being (SWN-20)**, M (SD)	85.5 (15.7)
**Antipsychotic medication**	
Dosage in mg/d of olanzapine equivalents, M (SD)	9.1 (4.8)
D_2_ profile, *N* (%)	
*High receptor affinity antagonism*	14 (25.0)
*Low receptor affinity antagonism*	27 (48.2)
*Partial agonism*	15 (26.8)

*Note*: FEP, first-episode psychosis.

a There were two missing values for this variable. For the regression analyses, these were complemented by data generated through multiple imputation using chained equations (MICE).

**Table 3.** Within-person distribution of momentary psychological side effects

Side effect	Mean (standard deviation)	Median (interquartile range)
**Blunted emotional experience**		
PA intensity	53.9 (16.7)	52.8 (41.1–63.1)
NA intensity	15.3 (17.3)	8.6 (2.3–22.4)
PA stability	8.5 (7.8)	6.3 (3.0–11.7)
NA stability	5.2 (6.3)	2.9 (1.1–6.6)
PA/NA variability	43.9 (25.2)	43.5 (23.2–62.6)
**Avolition**		
Initiative of social contact	46.0 (24.1)	51.6 (25.3–66.9)
Completion of daily life activities	56.8 (24.8)	54.9 (50.1–72.3)
**Mental fatigue**		
Tiredness/difficulty concentrating	57.1 (18.3)	56.2 (41.6–72.1)

*Note*: Each score except for PA/NA variability was rated on a visual analogue scale of 1–100. Note that higher scores indicate increased affect intensity and variability and more initiative of social contact and completion of daily life activities but less affect stability and mental fatigue. NA, negative affect; PA, positive affect.

### Regression analyses

#### Associations between D_2_ affinity and momentary psychological side effects

As compared with high-affinity D_2_ antagonists, the use of low-affinity D_2_ antagonists (B = 10.04 [95% CI: 0.59; 19.49], Table 4) and the use of partial D_2_ agonists (B = 12.98 [95% CI: 2.43; 23.53], Table 4) were associated with increased PA intensity. The difference in effects between low-affinity D_2_ antagonists versus partial D_2_ agonists was not statistically significant (B = 2.88 [95% CI: −6.94; 12.71]). There were no associations between D_2_ affinity and the other PSEs (Table 4). Except for the associations involving low-affinity D_2_ antagonists, results were largely consistent across different times of the day (Supplementary Materials 1–3).

**Table 4.** Multilevel mixed-effects linear regression analysis of associations between D_2_ affinity/dosage and momentary psychological side effects (*N* = 56; 3,005 observations)

	Coefficient beta (B) (95% confidence interval [CI])							
	Blunted emotional experience					Avolition		Mental fatigue
	PA intensity	NA intensity	PA stability	NA stability	PA/NA variability	Initiative of social contact	Completion of daily life activities	Tiredness/difficulty concentrating
Dosage	**−1.11** **[−1.97; −0.24]**	0.46 [−0.36; 1.27]	**0.23** **[0.04; 0.42]**	0.02 [−0.19; 0.22]	**−1.40** **[−2.52; −0.29]**	0.13 [−0.96; 1.21]	−0.70 [−1.60; 0.20]	−0.34 [−1.34; 0.66]
D_2_ group								
High affinity	Reference	Reference	Reference	Reference	Reference	Reference	Reference	Reference
Low affinity	**10.04** **[0.59; 19.49]**	−3.59 [−12.19; 5.01]	−0.60 [−2.47; 1.28]	−1.20 [−3.37; 0.97]	11.89 [−0.43; 24.21]	−2.74 [−14.26; 8.79]	−6.28 [−16.02; 3.46]	−0.90 [−10.86; 9.06]
Partial agonist	**12.98** **[2.43; 23.53]**	−2.03 [−13.36; 9.31]	−0.51 [−3.37; 2.35]	−1.40 [−3.90; 1.58]	11.13 [−3.20; 25.45]	−4.43 [−16.58; 7.72]	−3.71 [−15.59; 8.18]	0.69 [−13.49; 14.88]
ICC	0.55 [0.42; 0.68]	0.72 [0.59; 0.82]	0.02 [<0.01; 0.31]	0.32 [0.24; 0.42]	0.65 [0.52; 0.76]	0.40 [0.31; 0.50]	0.48 [0.38; 0.57]	0.63 [0.45; 0.78]

*Note*: Corrected for age, sex, symptom severity during first-episode psychosis (CASH), and current tobacco and cannabis use. Bold font indicates statistical significance at α = 0.05. Intraclass correlation coefficients (ICCs) originate from the final model, wherein either dosage alone or dosage and D_2_ group were included. Note that higher scores indicate increased affect intensity and variability and more initiative of social contact and completion of daily life activities, but less affect stability and mental fatigue.

#### Associations between dosage and momentary psychological side effects

**Figure 1.**
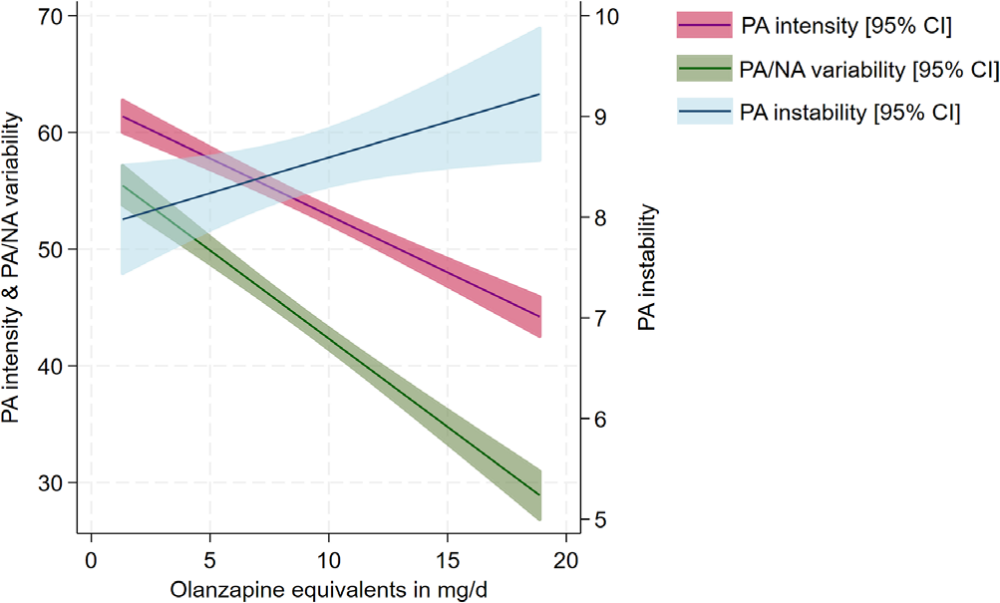
Positive affect (PA) intensity, positive and negative affect (PA/NA) variability and PA stability in relation to antipsychotic medication dosage (affect rated on visual analogue scales of 1–100).

A higher dosage of antipsychotic medication was associated with decreased intensity in PA (B = −1.11 [95% CI: −1.97; −0.24], Table 4, Figure 1), lower affect variability (B = −1.40 [95% CI: −2.52; −0.29], Table 4, Figure 1) as well as with greater instability in PA (B = 0.23 [95% CI: 0.04; 0.42], Table 4, Figure 1). Associations with PA stability were identified during the day and in the evenings but not in the mornings, while associations with affect variability were identified in the mornings and during the day but not in the evenings (Supplementary Materials 1–3). The ICCs in the models including PA intensity and affect variability were 0.55 (95% CI: 0.42; 0.68) (Table 4) and 0.65 (95% CI: 0.52; 0.76) (Table 4), respectively, while the ICC in the model including PA stability was 0.02 (95% CI: <0.01; 0.31) (Table 4). This indicates that intraindividual variation was relatively small in the former two and large in the latter model. There were no significant associations between dosage and NA intensity, NA stability, avolition, and mental fatigue (Table 4).

Analyses on the interaction between D_2_ affinity and dosage indicated that associations between dosage and concurrent levels of PSEs did not differ across D_2_-affinity profiles.

## Discussion

In the current EMA study, we investigated serial, cross-sectional associations between D_2_ affinity and dosage of antipsychotic medication and momentary mental states indicative of PSEs in 56 people in remission from FEP. Higher antipsychotic dosages were associated with reduced affect variability, lower PA stability, and decreased PA intensity. The latter was also associated with the use of high-affinity D_2_ antagonists.

Our analyses indicate that people who were taking haloperidol, risperidone, or amisulpride scored an average of 10–13 points lower on momentary PA (on a scale of 1–100) as compared with those taking olanzapine, quetiapine, aripiprazole, or brexpiprazole, and that a higher dosage could account for an additional reduction in PA, for example, 5.5 points for an increase equivalent to 5 mg olanzapine more per day. Furthermore, an increase of 5 mg olanzapine equivalents more per day was associated with a 7-point reduction in overall affect variability, indicating a measurable blunting of emotional experience associated with the use of antipsychotic medication. These results align with findings from an earlier EMA study in 109 people with chronic psychotic disorders showing that increased D_2_ occupancy was associated with lower PA in participants using high-affinity D_2_ antagonists (Lataster et al., 2011b). In addition, our data suggest that higher antipsychotic dosages may be linked with more instability in PA, a phenomenon that has previously been identified for overall affect in FEP and at-risk populations as compared with a healthy control group (Hermans et al., 2021). This could have implications with regard to altered emotional reactivity in psychotic disorders (Reininghaus et al., 2016), but the absence of associations with NA and the rather small effect size undermine the clinical relevance of those results. Taken together, the evidence may suggest that stronger D_2_ suppression by antipsychotic medication can lead to instable and generally lower levels of positive emotions, and that these might drive the frequently reported blunting of emotional experience (Bjornestad et al., 2020; Read & Sacia, 2020; Thompson et al., 2020; Wolters et al., 2003). Although the cross-sectional nature of our data precludes drawing causal conclusions, this interpretation is supported by an experimental randomized controlled trial wherein 85 healthy participants had increased levels of anhedonia under amisulpride compared with placebo (Berg, Riehle, Rief, & Lincoln, 2023). The results also align with the finding that a lower starting dosage of amisulpride was associated with a more favorable subjective response (de Haan et al., 2023) and corroborate the literature on the subjective tolerability of antipsychotic medication (Awad, 2019) indicating, for example, that >70% D_2_ receptor occupancy is associated with poorer subjective well-being (de Haan et al., 2003; Mizrahi et al., 2007).

Another HAMLETT study (de Beer et al., 2024) investigated associations between D_2_ affinity and clinically assessed negative symptoms at baseline after applying inverse probability of treatment weighting (i.e. thereby specifically addressing secondary negative symptoms). Participants using partial D_2_ agonists had significantly lower levels of secondary negative symptoms than those using D_2_ antagonists, and higher dosages were associated with more severe secondary negative symptoms across all D_2_-affinity profiles (de Beer et al., 2024). The similar observations from the current study support these findings by extending them to momentary affect as experienced in daily life.

It is worthwhile to note that the ICCs in the models investigating PA intensity and PA/NA variability indicate relatively large interindividual variation in their associations with antipsychotic dosages. This aligns with qualitative (Dijkstra et al., 2024) as well as quantitative (de Haan et al., 2023) studies showing that the subjective response to antipsychotic medication can vary considerably between patients. In contrast to the variation across individuals, results were relatively consistent across daytimes, albeit with the greatest effect sizes throughout the day and in the evenings and smaller effects in the beginning of the day (Supplementary Materials 1–3). This could reflect the usual time of taking the medication, which is in the evening for antagonists, and side effects usually being most severe within 1–4 hours after ingestion. Alternatively, it might reflect that PSEs of antipsychotic medication can be superimposed by other factors influencing affect, for example, diurnal rhythm-related upward trends in PA in the mornings and downward trends in the evenings (Murray et al., 2009) or possibly increased awareness of, or less distraction from, one’s emotional experiences in the evenings. Unfortunately, we do not have information on treatment compliance and timing of medication intake, which might have relevant implications for the interpretation of our findings, for example, in relation to the timing of peak plasma levels.

Considering our findings with respect to PA, it is somewhat unexpected that we found no association between the antipsychotic prescription and NA. This might suggest that dosage and D_2_ affinity are, in fact, distinctively associated with reduced experience of joy, or (consummatory) anhedonia (Visser et al., 2020). Indeed, levels of NA intensity were relatively low in our sample, indicating that depressive or anxiety-related mental states were, at most, of subclinical severity. This may have important implications for patients reporting reductions in their experience of pleasure, as anhedonia was found to impact personal recovery after FEP beyond symptomatic and functional recovery in another HAMLETT study (Maas et al., 2024). Overall, the current study contributes to mounting evidence highlighting a frequently overlooked psychological dimension of potential risks associated with antipsychotic-induced D_2_ suppression after FEP.

On a neurobiological level, our results could be explained by mesolimbic dopaminergic suppression (Juckel, 2016) with the potential risk of negative symptom exacerbation at commonly prescribed dosages (Sabe et al., 2021). Importantly, however, higher dosages were not significantly associated with any other PSEs we investigated. In fact, higher dosage and stronger D_2_ affinity were only associated with affective side effects but not with avolition or mental fatigue. One explanation might be that participants could have been less likely to complete the EMA questionnaire at moments when they experienced low motivation, difficulty concentrating or tiredness. Another explanation might lie in the concurrent use of other psychotropic agents or psychiatric comorbidity, but given the considerable variation in comorbidity and comedication among the relatively small number of affected participants, such an impact is likely to be minor. It is also possible that effects on those side effects are obscured by differences in simultaneous action on receptors such as histamine-1 (Fang et al., 2016), which are, however, highly variable among the currently used antipsychotic medications. For instance, low D_2_-affinity agents have antihistaminergic properties as well (albeit to varying degrees), while high D_2_-affinity agents generally do not (except for risperidone). Nevertheless, a study using clinical assessment methods in 520 people with schizophrenia (Fervaha et al., 2015) showed that sedative effects of antipsychotic medication were not associated with levels of motivation. Similarly, the authors found no association between motivation and the dosage or type of antipsychotic medication. It should be noted that dosages of antipsychotic medication in the HAMLETT baseline sample were generally low. Taken together, the current evidence indicates that levels of motivation in people who are in recent clinical remission from FEP are not associated with differences in the dosage or type of antipsychotic medications primarily targeting D_2_ receptors, at least when these agents are used in low dosages.

### Strengths and limitations

This is the first study to implement digital EMA to investigate associations between antipsychotic medication and PSEs in daily life after clinical remission from FEP. Therefore, our findings contribute to and extend the current body of evidence concerning the impact of antipsychotic medication on subjective well-being. Findings may be helpful in clinical practice with respect to decisions about dosing, type, and continuation of antipsychotic medication after FEP (Karow et al., 2007). A particular strength is that our findings are based on a large number of data points originating from various daily life situations, including work as well as leisure contexts (Djordjevic et al., 2025), and were relatively equally distributed throughout the day, indicating valid representation of people’s daily life.

There are also limitations to our study. First, despite correction for severity of FEP based on the CASH, we cannot entirely exclude the possibility of bias related to confounding by indication (e.g. higher dosages for more severe illness). Nevertheless, symptom severity at the time of daily life assessments can be expected to have had, at most, a minimal impact on prescription patterns, as none of the participants had clinically relevant psychotic symptoms at the time of study inclusion. Second, due to the naturalistic study design, the medication groups were not equal in size and some associations between D_2_ affinity and PSEs might have therefore been missed. Similarly, future investigations will need to include larger and more diverse samples, for example, with respect to initial treatment response. Third, since our sample comprises only around one-fifth of all HAMLETT participants, the results may not be entirely representative of the total sample. Importantly, however, a recent study including the non-EMA HAMLETT sample identified similar patterns in associations between antipsychotic prescriptions and secondary negative symptoms as assessed through clinical questionnaires (de Beer et al., 2024), indicating that our results are most probably not specific to the EMA sample. Finally, although our questionnaire length and assessment frequency align with established EMA research protocols in psychotic disorders (Bell et al., 2023), there may be bias related to fatigue or habituation effects (Ulitzsch et al., 2025). However, given that completion rates and response times were consistent with previous work (Bell et al., 2023), the risk of such bias is small. Moreover, it is improbable that the respective bias would differ between D_2_ affinities.

## Conclusion

In sum, the current study suggests that, after clinical remission from FEP, a higher antipsychotic dosage and the use of high-affinity D_2_ antagonists, as compared with partial D_2_ agonists or low-affinity D_2_ antagonists, are associated with flattened momentary affects and decreased PA in particular, but not with avolition or mental fatigue as estimated with EMA. It also suggests that blunted emotional experience associated with higher antipsychotic dosages might be mainly driven by those reductions in PA. The identified associations were largely consistent across different times of the day, though effect sizes related to PA were smallest in the mornings and greatest in the evenings. Future research using longitudinal EMA data of PSEs is warranted, since replication and extension of our results harbor implications for the medical management of psychotic disorders.

## Supporting information

Djordjevic et al. supplementary material Djordjevic et al. supplementary material

## References

[r1] Andreasen, N. C., Carpenter, W. T., Kane, J. M., Lasser, R. A., Marder, S. R., & Weinberger, D. R. (2005). Remission in schizophrenia: Proposed criteria and rationale for consensus. American Journal of Psychiatry: Official Journal of the American Psychiatric Association, 162(3), 441–449. 10.1176/appi.ajp.162.3.441. 15741458

[r2] Andreasen, N. C., Flaum, M., & Arndt, S. (1992) The Comprehensive Assessment of Symptoms and History (CASH): An Instrument for Assessing Diagnosis and Psychopathology. Arch Gen Psychiatry. 49(8), 615–623. 10.1001/archpsyc.1992.01820080023004. 1637251

[r3] Awad, A. G. (2019). Revisiting the concept of subjective tolerability to antipsychotic medications in schizophrenia and its clinical and research implications: 30 years later. CNS Drugs, 33(1), 1–8. 10.1007/s40263-018-0588-3. 30511350

[r4] Begemann, M. J. H., Thompson, I. A., Veling, W., Gangadin, S. S., Geraets, C. N. W., van ’t Hag, E., Müller-Kuperus, S. J., Oomen, P. P., Voppel, A. E., van der Gaag, M., Kikkert, M. J., Van Os, J., Smit, H. F. E., Knegtering, R. H., Wiersma, S., Stouten, L. H., Gijsman, H. J., Wunderink, L., Staring, A. B. P., … Sommer, I. E. C. (2020). To continue or not to continue? Antipsychotic medication maintenance versus dose-reduction/discontinuation in first episode psychosis: HAMLETT, a pragmatic multicenter single-blind randomized controlled trial. Trials 21, 147. 10.1186/s13063-019-3822-5. 32033579 PMC7006112

[r5] Bell, I. H., Eisner, E., Allan, S., Cartner, S., Torous, J., Bucci, S., & Thomas, N. (2023). Methodological characteristics and feasibility of ecological momentary assessment studies in psychosis: a systematic review and meta-analysis. Schizophrenia Bulletin, 50(2), 238–265. 10.1093/schbul/sbad127. PMC10919779 37606276

[r6] Berg, M., Riehle, M., Rief, W., & Lincoln, T. (2023). Does partial blockade of dopamine D2 receptors with Amisulpride cause anhedonia? An experimental study in healthy volunteers. Journal of Psychiatric Research, 158, 409–416. 10.1016/j.jpsychires.2023.01.014. 36680855

[r7] Bjornestad, J., Lavik, K. O., Davidson, L., Hjeltnes, A., Moltu, C., & Veseth, M. (2020). Antipsychotic treatment – A systematic literature review and meta-analysis of qualitative studies. Journal of Mental Health, 29(5), 513–523. 10.1080/09638237.2019.1581352. 30862219

[r8] Bobes, J., Arango, C., Garcia-Garcia, M., Rejas, J., & CLAMORS Study Collaborative Group. (2010). Prevalence of negative symptoms in outpatients with schizophrenia spectrum disorders treated with antipsychotics in routine clinical practice: findings from the CLAMORS study. The Journal of Clinical Psychiatry, 71(3), 280–286. 10.4088/JCP.08m04250yel. 19895779

[r9] Bos, E. H., de Jonge, P., & Cox, R. F. A. (2019). Affective variability in depression: Revisiting the inertia–instability paradox. British Journal of Psychology, 110(4), 814–827. 10.1111/bjop.12372. 30588616 PMC6899922

[r10] Bos, F. M., Schoevers, R. A., & aan het Rot, M. (2015). Experience sampling and ecological momentary assessment studies in psychopharmacology: A systematic review. European Neuropsychopharmacology, 25(11), 1853–1864. 10.1016/j.euroneuro.2015.08.008. 26336868

[r11] Ceraso, A., Lin, J. J., Schneider-Thoma, J., Siafis, S., Tardy, M., Komossa, K., Heres, S., Kissling, W., Davis, J. M., & Leucht, S. (2020). Maintenance treatment with antipsychotic drugs for schizophrenia. Cochrane Database of Systematic Reviews, 8. CD008016. DOI: 10.1002/14651858.CD008016.pub3. 32840872 PMC9702459

[r12] Correll, C. U., & Schooler, N. R. (2020). Negative symptoms in schizophrenia: A review and clinical guide for recognition, assessment, and treatment. Neuropsychiatric Disease and Treatment, 16, 519–534. 10.2147/ndt.S225643. 32110026 PMC7041437

[r13] de Beer, F., Wijnen, B., Wouda, L., Koops, S., Gangadin, S., Veling, W., van Beveren, N., de Haan, L., Begemann, M. J. H., & Sommer, I. E. C. (2024). Antipsychotic dopamine D2 affinity and negative symptoms in remitted first episode psychosis patients. Schizophrenia Research, 274, 299–306. 39426016 10.1016/j.schres.2024.09.030

[r14] de Haan, L., Lavalaye, J., Linszen, D., Dingemans, P. M. A. J., & Booij, J. (2000). Subjective experience and striatal dopamine D2 receptor occupancy in patients with schizophrenia stabilized by olanzapine or risperidone. American Journal of Psychiatry, 157(6), 1019–1020. 10.1176/appi.ajp.157.6.1019. 10831489

[r15] de Haan, L., van Bruggen, M., Lavalaye, J., Booij, J., Dingemans, P. M. A. J., & Linszen, D. (2003). Subjective experience and D2 receptor occupancy in patients with recent-onset schizophrenia treated with low-dose olanzapine or haloperidol: A randomized, double-blind study. American Journal of Psychiatry, 160(2), 303–309. 10.1176/appi.ajp.160.2.303. 12562577

[r16] de Haan, L., van Tricht, M., van Dijk, F., Arango, C., Díaz-Caneja, C. M., Bobes, J., García-Álvarez, L., & Leucht, S. (2023). Optimizing subjective wellbeing with amisulpride in first episode schizophrenia or related disorders. Psychological Medicine, 53(13), 5986–5991. 10.1017/S0033291722003142. 36520136 PMC10520587

[r17] Delespaul, P. A. E. G. (1995). *Assessing schizophrenia in daily life: The experience sampling method* UPM, Universitaire Pers Maastricht. WorldCat. Maastricht.

[r18] Dijkstra, S. A., Rijkeboer, J., Noordhof, A., Boyette, L.-L., Berendsen, S., de Koning, M., Bennen, R. L. J., Hofman, T., & de Haan, L. (2024). Making sense of recovery from first psychosis with antipsychotic medication: A qualitative phenomenological study. Schizophrenia Bulletin, 50(6), 1508–1520. 10.1093/schbul/sbae104. 39004928 PMC11548922

[r19] Djordjevic, M., Jongsma, H. E., Simons, C. J. P., Oomen, P. P., de Haan, L., Boonstra, N., Kikkert, M., Koops, S., Geraets, C. N. W., Begemann, M. J. H., Marcelis, M., & Veling, W. (2025). Associations between momentary mental states and concurrent social functioning after remission from first episode psychosis: A HAMLETT ecological momentary assessment study. Journal of Psychiatric Research, 181, 560–569. 10.1016/j.jpsychires.2024.12.002. 39708772

[r21] Fang, F., Sun, H., Wang, Z., Ren, M., Calabrese, J. R., & Gao, K. (2016). Antipsychotic drug-induced somnolence: Incidence, mechanisms, and management. CNS Drugs, 30(9), 845–867. 10.1007/s40263-016-0352-5. 27372312

[r22] Fervaha, G., Takeuchi, H., Lee, J., Foussias, G., Fletcher, P. J., Agid, O., & Remington, G. (2015). Antipsychotics and Amotivation. Neuropsychopharmacology, 40(6), 1539–1548. 10.1038/npp.2015.3. 25567425 PMC4397414

[r23] Granholm, E., Holden, J. L., Mikhael, T., Link, P. C., Swendsen, J., Depp, C., Moore, R. C., & Harvey, P. D. (2019). What do people with schizophrenia do all day? Ecological momentary assessment of real-world functioning in schizophrenia. Schizophrenia Bulletin, 46(2), 242–251. 10.1093/schbul/sbz070. PMC7442321 31504955

[r24] Haddad, P. M., Das, A., Keyhani, S., & Chaudhry, I. B. (2012). Antipsychotic drugs and extrapyramidal side effects in first episode psychosis: A systematic review of head–head comparisons. Journal of Psychopharmacology, 26(5_suppl), 15–26. 10.1177/0269881111424929 22057019

[r25] Hermans, K. S. F. M., Myin-Germeys, I., Gayer-Anderson, C., Kempton, M. J., Valmaggia, L., McGuire, P., Murray, R. M., Garety, P., Wykes, T., Morgan, C., Kasanova, Z., & Reininghaus, U. (2021). Elucidating negative symptoms in the daily life of individuals in the early stages of psychosis. Psychological Medicine, 51(15), 2599–2609. 10.1017/S0033291720001154. 32438944 PMC8579154

[r26] Horwitz, A. G., Zhao, Z., & Sen, S. (2023). Peak-end bias in retrospective recall of depressive symptoms on the PHQ-9. Psychological Assessment 10.1037/pas0001219 PMC10052790 36757996

[r27] Juckel, G. (2016). Inhibition of the reward system by antipsychotic treatment. Dialogues in Clinical Neuroscience, 18(1), 109–114. 10.31887/DCNS.2016.18.1/gjuckel. 27069385 PMC4826766

[r28] Kaar, S. J., Natesan, S., McCutcheon, R., & Howes, O. D. (2020). Antipsychotics: Mechanisms underlying clinical response and side-effects and novel treatment approaches based on pathophysiology. Neuropharmacology, 172, 107704. 10.1016/j.neuropharm.2019.107704. 31299229

[r29] Kahn, R. S., Fleischhacker, W. W., Boter, H., Davidson, M., Vergouwe, Y., Keet, I. P. M., Gheorghe, M. D., Rybakowski, J. K., Galderisi, S., Libiger, J., Hummer, M., Dollfus, S., López-Ibor, J. J., Hranov, L. G., Gaebel, W., Peuskens, J., Lindefors, N., Riecher-Rössler, A., & Grobbee, D. E. (2008). Effectiveness of antipsychotic drugs in first-episode schizophrenia and schizophreniform disorder: an open randomised clinical trial. The Lancet, 371(9618), 1085–1097. 10.1016/S0140-6736(08)60486-9. 18374841

[r30] Karow, A., Czekalla, J., Dittmann, R. W., Schacht, A., Wagner, T., Lambert, M., Schimmelmann, B. G., & Naber, D. (2007). Association of subjective well-being, symptoms, and side effects with compliance after 12 months of treatment in schizophrenia. The Journal of Clinical Psychiatry, 68(1), 75–80. 10.4088/jcp.v68n0110. 17284133

[r31] Kay S.R., Fiszbein, A., & Opler, L. A. (1987) The Positive and Negative Syndrome Scale (PANSS) for Schizophrenia. Schizophrenia Bulletin, 13(2), 261–276, 10.1093/schbul/13.2.261. 3616518

[r32] Kirschner, M., Aleman, A., & Kaiser, S. (2017). Secondary negative symptoms — A review of mechanisms, assessment and treatment. Schizophrenia Research, 186, 29–38. 10.1016/j.schres.2016.05.003. 27230288

[r33] Kumari, V., & Postma, P. (2005). Nicotine use in schizophrenia: The self medication hypotheses. Neuroscience & Biobehavioral Reviews, 29(6), 1021–1034. 10.1016/j.neubiorev.2005.02.006. 15964073

[r34] Lataster, J., Myin-Germeys, I., Wichers, M., Delespaul, P. A. E. G., van Os, J., & Bak, M. (2011a). Psychotic exacerbation and emotional dampening in the daily life of patients with schizophrenia switched to aripiprazole therapy: a collection of standardized case reports. Therapeutic Advances in Psychopharmacology, 1(5), 145–151. 10.1177/2045125311419552. 23983939 PMC3736906

[r35] Lataster, J., van Os, J., de Haan, L., Thewissen, V., Bak, M., Lataster, T., Lardinois, M., Delespaul, P. A. G. E., & Myin-Germeys, I. (2011b). Emotional experience and estimates of D2 receptor occupancy in psychotic patients treated with haloperidol, risperidone, or olanzapine: An experience sampling study. The Journal of Clinical Psychiatry, 72(10), 1397–1404. 10.4088/JCP.09m05466yel. 21208588

[r36] Leucht, S., Crippa, A., Siafis, S., Patel, M. X., Orsini, N., & Davis, J. M. (2020). Dose-response meta-analysis of antipsychotic drugs for acute schizophrenia. American Journal of Psychiatry, 177(4), 342–353. 10.1176/appi.ajp.2019.19010034. 31838873

[r37] Longden, E., & Read, J. (2016). Assessing and reporting the adverse effects of antipsychotic medication: A systematic review of clinical studies, and prospective, retrospective, and cross-sectional research. Clinical Neuropharmacology, 39(1), 29–39. 10.1097/wnf.0000000000000117. 26757311

[r38] Maas, I. L., Bohlken, M. M., Gangadin, S. S., Rosema, B.-S., Veling, W., Boonstra, N., de Haan, L., Begemann, M. J. H., & Koops, S. (2024). Personal recovery in first-episode psychosis: Beyond clinical and functional recovery. Schizophrenia Research, 266, 32–40. 10.1016/j.schres.2024.02.005. 38367610

[r39] Mas, S., Gassó, P., de Bobadilla, R., Arnaiz, J. A., Bernardo, M., & Lafuente, A. (2013). Secondary nonmotor negative symptoms in healthy volunteers after single doses of haloperidol and risperidone: A double-blind, crossover, placebo-controlled trial. Human Psychopharmacology: Clinical and Experimental, 28(6), 586–593. 10.1002/hup.2350. 24519692

[r40] Mizrahi, R., Rusjan, P., Agid, O., Graff, A., Mamo, D. C., Zipursky, R. B., & Kapur, S. (2007). Adverse subjective experience with antipsychotics and its relationship to striatal and Extrastriatal D 2 receptors: A PET study in schizophrenia. American Journal of Psychiatry, 164(4), 630–637. 10.1176/ajp.2007.164.4.630. 17403977

[r41] Mote, J., & Fulford, D. (2020). Ecological momentary assessment of everyday social experiences of people with schizophrenia: A systematic review. Schizophrenia Research, 216, 56–68. 10.1016/j.schres.2019.10.021. 31874743

[r42] Murray, G., Nicholas, C. L., Kleiman, J., Dwyer, R., Carrington, M. J., Allen, N. B., & Trinder, J. (2009). Nature’s clocks and human mood: The circadian system modulates reward motivation. Emotion (Washington, D.C.), 9(5), 705–716. 10.1037/a0017080. 19803592

[r43] Myin-Germeys, I., Oorschot, M., Collip, D., Lataster, J., Delespaul, P., & van Os, J. (2009). Experience sampling research in psychopathology: Opening the black box of daily life. Psychological Medicine, 39(9), 1533–1547. 10.1017/s0033291708004947. 19215626

[r44] Myin-Germeys, I., & van Os, J. (2007). Stress-reactivity in psychosis: Evidence for an affective pathway to psychosis. Clinical Psychology Review, 27(4), 409–424. 10.1016/j.cpr.2006.09.005. 17222489

[r45] Naber, D. (1995). A self-rating to measure subjective effects of neuroleptic drugs, relationships to objective psychopathology, quality of life, compliance and other clinical variables. International Clinical Psychopharmacology, 10, 133–138. 10.1097/00004850-199509000-00017. 8866775

[r46] National Institute for Health and Care Excellence (NICE). (2014). National Clinical Guideline Number 178: Psychosis and Schizophrenia in Adults. National Institute for Health and Care Excellence, London, UK.

[r47] Oomen, P. P., Simons, C. J. P., Broekmans-Madikrama, K., & Marcelis, M. (2024). Monitoring momentary subjective well-being and psychotic experiences during antipsychotic dose reduction: Two single-case time series experience sampling method pilot study. Psychiatric Rehabilitation Journal, 47(4), 329–338. 10.1037/prj0000621. 39052408

[r48] Oorschot, M., Kwapil, T., Delespaul, P., & Myin-Germeys, I. (2009). Momentary assessment research in psychosis. Psychological Assessment, 21(4), 498–505. 10.1037/a0017077. 19947784

[r49] Orygen. (2010). Australian clinical guidelines for early psychosis.

[r50] Rashmi, P., Robin, W., Richard, J., Michael, B., Hitesh, S., Matthew, B., Robert, S., Philip, M., & Sagnik, B. (2016). Association of cannabis use with hospital admission and antipsychotic treatment failure in first episode psychosis: an observational study. BMJ Open, 6(3), e009888. 10.1136/bmjopen-2015-009888. PMC4785290 26940105

[r51] Read, J., & Sacia, A. (2020). Using open questions to understand 650 people’s experiences with antipsychotic drugs. Schizophrenia Bulletin, 46(4), 896–904. 10.1093/schbul/sbaa002. 32047917 PMC7345822

[r52] Reininghaus, U., Kempton, M. J., Valmaggia, L., Craig, T. K. J., Garety, P., Onyejiaka, A., Gayer-Anderson, C., So, S. H., Hubbard, K., Beards, S., Dazzan, P., Pariante, C., Mondelli, V., Fisher, H. L., Mills, J. G., Viechtbauer, W., McGuire, P., van Os, J., Murray, R. M., … Morgan, C. (2016). Stress sensitivity, aberrant salience, and threat anticipation in early psychosis: An experience sampling study. Schizophrenia Bulletin, 42(3), 712–722. 10.1093/schbul/sbv190. 26834027 PMC4838104

[r53] Sabe, M., Zhao, N., Crippa, A., & Kaiser, S. (2021). Antipsychotics for negative and positive symptoms of schizophrenia: dose-response meta-analysis of randomized controlled acute phase trials. NPJ Schizophrenia, 7(1), 43. 10.1038/s41537-021-00171-2. 34518532 PMC8438046

[r54] StataCorp. (2021). *Stata Statistical Software: Release 17.* In StataCorp LLC.

[r55] Thompson, J., Stansfeld, J. L., Cooper, R. E., Morant, N., Crellin, N. E., & Moncrieff, J. (2020). Experiences of taking neuroleptic medication and impacts on symptoms, sense of self and agency: a systematic review and thematic synthesis of qualitative data. Social Psychiatry and Psychiatric Epidemiology, 55(2), 151–164. 10.1007/s00127-019-01819-2. 31875238

[r63] Ulitzsch, E., Viechtbauer, W., Lüdtke, O., Myin-Germeys, I., Nagy, G., Nestler, S., & Eisele, G. V. (2025). Investigating the effect of experience sampling study design on careless and insufficient effort responding identified with a screen-time-based mixture model. Psychological Assessment, 37(8), 347–359. 10.1037/pas0001379 40338562

[r56] Urban, E. J., Charles, S. T., Levine, L. J., & Almeida, D. M. (2018). Depression history and memory bias for specific daily emotions. PLoS One, 13(9), e0203574. 10.1371/journal.pone.0203574. 30192853 PMC6128594

[r57] Veerman, S. R. T., Schulte, P. F. J., & de Haan, L. (2017). Treatment for negative symptoms in schizophrenia: A comprehensive review. Drugs, 77(13), 1423–1459. 10.1007/s40265-017-0789-y. 28776162

[r58] Veling, W., Boonstra, N., van Doorne, H., de Haan, L., van der Gaag, M., Gijsman, H. J., Kamstra, D., Klaassen, R., Kleijwegt, H., & Lansen, M. (2017). Zorgstandaard psychose, module Vroege Psychose. Netwerk Kwaliteitsontwikkeling GGZ.

[r59] Visser, K. F., Chapman, H. C., Ruiz, I., Raugh, I. M., & Strauss, G. P. (2020). A meta-analysis of self-reported anticipatory and consummatory pleasure in the schizophrenia-spectrum. Journal of Psychiatric Research, 121, 68–81. 10.1016/j.jpsychires.2019.11.007. 31783235 PMC6939125

[r60] Wolters, H. A., Knegtering, R., Wiersma, D., & Van Den Bosch, R. J. (2003). The spectrum of subjective effects of antipsychotic medication. Acta Neuropsychiatrica, 15(5), 274–279. 10.1034/j.1601-5215.2003.00038.x 26983656

[r61] Wright, A. C., Browne, J., Skiest, H., Bhiku, K., Baker, J. T., & Cather, C. (2021). The relationship between conventional clinical assessments and momentary assessments of symptoms and functioning in schizophrenia spectrum disorders: A systematic review. Schizophrenia Research, 232, 11–27. 10.1016/j.schres.2021.04.010. 34004382

[r62] Zhang, J.-P., Gallego, J. A., Robinson, D. G., Malhotra, A. K., Kane, J. M., & Correll, C. U. (2013). Efficacy and safety of individual second-generation vs. first-generation antipsychotics in first-episode psychosis: A systematic review and meta-analysis. International Journal of Neuropsychopharmacology, 16(6), 1205–1218. 10.1017/s1461145712001277. 23199972 PMC3594563

