# Supplementary material for: Psychological side effects of antipsychotic medication after remission from first-episode psychosis: a HAMLETT ecological momentary assessment study
Source: Psychol Med. 2025 Oct 29;55:e325. doi: 10.1017/S003329172510216X (PMC13054896; doi:10.1017/S003329172510216X)
Supplement: Djordjevic et al. supplementary material [file S003329172510216Xsup001.docx]

**Supplementary material**

Supplementary Material 1: Multilevel mixed-effects linear regression analysis of associations between D_2_ affinity /dosage and momentary psychological side-effects in the beginning of the day (prompts 1-3, N=873 observations)

|  | *Coefficient Beta (B) [95% Confidence Interval (CI)]* | | | | | | | |
| --- | --- | --- | --- | --- | --- | --- | --- | --- |
|  | Blunted emotional experience | | | | | Avolition | | Mental fatigue |
|  | PA intensity | NA intensity | PA stability | NA stability | PA/NA variability | Initiative of social contact | Completion of daily life activities | Tiredness/difficulty concentrating |
| Dosage | -**0.89**  **[-1.74; -0.03]** | 0.49  [-0.35; 1.34] | 0.10  [-0.13; 0.32] | 0.06  [-0.17; 0.30] | **-1.23**  **[-2.36; -0.10]** | 0.16  [-0.97; 1.29] | -0.86  [-1.83; 0.10] | -0.29  [-1.32; 0.73] |
| D_2_ group  High affinity  Low affinity  Partial agonist | Reference  7.80  [-1.48; 17.08]  **12.08**  **[1.38; 22.78]** | Reference  -4.73  [-13.74; 4.28]  -2.31  [-13.83; 9.22] | Reference  -0.30  [-2.42; 1.82]  -0.13  [-3.33; 3.06] | Reference  -0.84  [-3.23; 1.55]  -1.06  [-4.14; 2.01] | Reference  9.31  [-3.08; 21.70]  10.25  [-4.27; 24.76] | Reference  -4.83  [-16.59; 6.93]  -6.52  [-19.13; 6.09] | Reference  -7.68  [-17.18; 1.81]  -4.33  [-16.33; 7.67] | Reference  -3.35  [-13.08; 6.38]  -1.16  [-15.73; 13.40] |

Corrected for age, sex, symptom severity during first episode psychosis (CASH) and current tobacco and cannabis use. Bold font indicates statistical significance at α=0.05. Note that higher scores indicate increased affect intensity and variability and more initiative of social contact and completion of daily life activities but less affect stability and mental fatigue.

Supplementary Material 2: Multilevel mixed-effects linear regression analysis of associations between D_2_ affinity /dosage and momentary psychological side-during daytime (prompts 4-7, N=1256 observations)

|  | *Coefficient Beta (B) [95% Confidence Interval (CI)]* | | | | | | | |
| --- | --- | --- | --- | --- | --- | --- | --- | --- |
|  | Blunted emotional experience | | | | | Avolition | | Mental fatigue |
|  | PA intensity | NA intensity | PA stability | NA stability | PA/NA variability | Initiative of social contact | Completion of daily life activities | Tiredness/difficulty concentrating |
| Dosage | **-1.27**  **[-2.16; -0.38]** | 0.45  [-0.37; 1.28] | **0.26**  **[0.05; 0.48]** | -0.03  [-0.25; 0.19] | **-1.51**  **[-2.66; -0.37]** | 0.07  [-1.06; 1.19] | -0.69  [-1.64; 0.26] | -0.49  [-1.53; 0.55] |
| D_2_ group  High affinity  Low affinity  Partial agonist | Reference  **10.80**  **[1.14; 20.46]**  **12.01**  **[1.29; 22.73]** | Reference  -3.42  [-11.92; 5.08]  -1.84  [-13.34; 9.66] | Reference  -1.36  [-3.14; 0.41]  **-2.58**  **[-4.88; -0.29]** | Reference  -1.61 [-3.95; 0.72]  -1.79 [-4.61; 1.02] | Reference  12.49  [-0.05; 25.03]  10.27  [-4.35; 24.89] | Reference  -1.18  [-12.95; 10.59]  -5.32  [-17.65; 7.01] | Reference  -5.07  [-15.29; 5.14]  -3.87  [-16.53; 8.79] | Reference  0.02  [-10.65; 10.69]  -1.03  [-15.55; 13.48] |

Corrected for age, sex, symptom severity during first episode psychosis (CASH) and current tobacco and cannabis use. Bold font indicates statistical significance at α=0.05. Note that higher scores indicate increased affect intensity and variability and more initiative of social contact and completion of daily life activities but less affect stability and mental fatigue.

Supplementary Material 3: Multilevel mixed-effects linear regression analysis of associations between D_2_ affinity /dosage and momentary psychological side-at the end of the day (prompts 8-10, N=876 observations)

|  | *Coefficient Beta (B) [95% Confidence Interval (CI)]* | | | | | | | |
| --- | --- | --- | --- | --- | --- | --- | --- | --- |
|  | Blunted emotional experience | | | | | Avolition | | Mental fatigue |
|  | PA intensity | NA intensity | PA stability | NA stability | PA/NA variability | Initiative of social contact | Completion of daily life activities | Tiredness/difficulty concentrating |
| Dosage | **-1.04**  **[-1.95; -0.13]** | 0.46  [-0.33; 1.26] | **0.29**  **[0.06; 0.53]** | 0.02  [-0.16; 0.21] | -1.46  [-3.77; 0.85] | 0.24  [-0.92; 1.39] | -0.59  [-1.53; 0.35] | -0.10  [-1.13; 0.92] |
| D_2_ group  High affinity  Low affinity  Partial agonist | Reference  **11.57**  **[1.65; 21.49]**  **14.94**  **[3.97; 25.92]** | Reference  -2.41  [-10.68; 5.87]  -1.17  [-12.23; 9.88] | Reference  0.36  [-2.07; 2.78]  1.04  [-2.07; 4.15] | Reference  -1.00  [-2.93; 0.93]  -0.43  [-2.95; 2.09] | Reference  10.91  [-14.09; 35.91]  12.45  [-15.95; 40.85] | Reference  -2.58  [-14.99; 9.82]  -1.94  [-15.43; 11.54] | Reference  -7.40  [-17.56; 2.76]  -3.21  [-15.49; 9.06] | Reference  0.31  [-9.54; 10.17]  4.45  [-9.50; 18.41] |

Corrected for age, sex, symptom severity during first episode psychosis (CASH) and current tobacco and cannabis use. Bold font indicates statistical significance at α=0.05. Note that higher scores indicate increased affect intensity and variability and more initiative of social contact and completion of daily life activities but less affect stability and mental fatigue.
